# Evaluating Different Aspects of Prospective Memory in Amnestic and Nonamnestic Mild Cognitive Impairment

**DOI:** 10.1155/2014/805929

**Published:** 2014-03-05

**Authors:** Rene Hernandez Cardenache, Lizmar Burguera, Amarilis Acevedo, Rosie Curiel, David A. Loewenstein

**Affiliations:** ^1^Department of Psychiatry and Behavioral Sciences, Miller School of Medicine, University of Miami, FL 33134, USA; ^2^Department of Psychology, Nova Southeastern University, Davie, FL 33314, USA; ^3^Center on Aging, Miller School of Medicine, University of Miami, FL 33134, USA

## Abstract

Prospective memory, the inability to remember an intended action, is a common complaint, but not formally assessed in most clinical and research studies of mild cognitive impairment (MCI). In this study, patients with amnestic mild cognitive impairment (aMCI), non-amnestic cognitive impairment (naMCI), and cognitively normal (CN) elders were assessed using the Miami Prospective Memory Test (MPMT). A unique aspect of the paradigm was that participants were scored for intention to perform, accuracy in recollection for specific elements of the task, and the need for reminder cues. Excellent test-retest stability was obtained for MPMT Event-Related (ER), combined Time-Related (TR) subscales, and total MPMT score for aMCI subjects. MPMT impairments were observed in 48.6% of aMCI, 29.4% of naMCI, and 10.0% of normal elderly participants. Prospective memory deficits were common in participants with aMCI, and occurred in almost a third of naMCI participants. Intention to perform and need for reminder cues were significantly more impaired than retrospective memory for specific details of the task. It is concluded that assessment of different elements of prospective memory is important in MCI research and that inability to remember intended actions is a significant feature in those as risk for Alzheimer's disease.

## 1. Introduction 

Amnestic mild cognitive impairment (aMCI) has increasingly been accepted as a prodrome or significant risk factor for Alzheimer's disease in clinical settings [1]. The vast majority of efforts to assess aMCI have relied on paradigms that focus on retrospective memory. These involve typical list learning tests or measuring episodic memory for passages or visual reproduction tasks. Impairments in delayed recall or rate of forgetting on verbal episodic memory tasks have been found to be a sensitive indicator of mild Alzheimer's disease (AD) [2, 3] and a predictor of progression to dementia among elders who do not meet criteria for dementia upon initial evaluation [4–6].

Despite these efforts, with the growing understanding that earlier treatments may lead to better outcomes, there is a pressing need to develop tests that are optimally sensitive to different types of memory deficits in the earliest stages of neurodegenerative disorders such as Alzheimer's disease. Previous memory models have relied on retrospective memory (RM) (i.e., a type of episodic memory), involving remembering events experienced in one's past. Prospective memory (PM) is another form of episodic memory defined as remembering to carry out intended actions at an appropriate time in the future [7]. It is understood as a process of “remembering to remember” and is an integral aspect of episodic memory, most closely involving the formation, maintenance, and execution of future intentions. The construct of prospective memory can be further delineated by event-based PM, and time-based PM functions. Prospective memory is usually evaluated by requiring a patient/subject to perform an action either upon the occurrence of specified event (i.e., event-based PM task) or after a designated amount of time has elapsed (i.e., time-based PM task), while the patient is engaged in ongoing activity.

PM deficits have been widely observed in mild traumatic brain injury (mTBI) and are frequently observed in the absence of retrospective memory deficits; see [8, 9]. More recently, there has been increasing evidence that PM deficits are observed in subjects with aMCI [10–12].

One of the most widely used measures of prospective memory has been the Rivermead Behavioral Battery [13], which has several tasks, which assess prospective memory. One task is to remember to ask the examiner a couple of questions after the ringing of a bell, while the other is to have an examiner provide two objects which the examinee must later ask for and tell the examiner where they have been hidden. Another measure, the Memory for Intentions Test [14] assesses the examinee's ability to perform simple verbal or performance-based prospective memory tasks (e.g., writing ones name when given a red pen) which may vary with regards to time interval and whether prompting is involved. Jones et al. [15] developed a measure that requires the examinee to remember to make a request of the examiner at the end of their session together. A correct response was recorded whether the participant spontaneously remembered the task or a cue had to be provided. Unfortunately, this task was so difficult that the successful completion was attained by only a third of normal elderly participants.

Limitations of previous PM paradigms are as follows. First, the tasks are relatively simple and do not involve multistep components that reflect the complexity of real-world demands. For example, an individual may have to remember an intended action such as a doctor's appointment but may also have to remember to arrive 30 minutes early to do paper work and to bring in all medications. Another limitation of previous prospective memory tests is that they do not provide the means by which to compare the relative contributions of memory for intentions, accuracy, and ability to respond to prompts and reminders. The ability to examine these distinct components in the context of a time or event-related prospective memory task could have considerable advantages in a clinical or research setting. A final issue with existing prospective memory tests is whether an older adult with auditory or attentional issues may fully understand the task, which they are supposed to perform.

To this end, the purpose of the current investigation was to establish initial test-retest reliability and discriminative validity of a newly developed prospective memory test which was sufficiently complex, so that the effects of prospective memory, response to cuing, and accuracy of the responses could each be scaled to measure different degrees of proficiency on different event and time-based PMT tasks. It was our intention to determine the degree to which event-related and time-related prospective memory abilities differed in the assessment of amnestic MCI (aMCI), nonamnestic MCI (na-MCI), and cognitively normal (CN) elders.

## 2. Methods 

### 2.1. Subjects

We recruited different subsets of subjects from a study investigating longitudinal changes associated with mild cognitive impairment and normal aging. In addition, subjects were recruited from the memory disorders clinic at the Wien Center for Alzheimer's Disease and Memory Disorders at Mount Sinai Medical Center and the community as described below. Subjects diagnosed with amnestic mild cognitive impairment (aMCI) met Petersen's [16] criteria. This includes a memory complaint by the patient and preferably an informant, objective memory deficits on clinical evaluation and cognitive deficits not sufficient to interfere with social and/or occupational functioning as defined by DSM-IV-TR criteria (American Psychiatric Association, 2000). All of these subjects obtained a global Clinical Dementia Rating (CDR) Score [17] of .5, equivalent to MCI, and had memory impairment at 1.5 SD or greater below expected levels on the total recall of the Fuld Object Memory Evaluation [18]; Delayed Logical Memory or Delayed Visual Reproduction of the WMS-III [19].

We evaluated participants who met Petersen's [16] criteria for nonamnestic MCI, all of whom had a CDR global score of .5, nonimpaired scores on memory measures described above, but scored 1.5 SD or lower on one or more nonamnestic measures such as letter fluency [20], category fluency [20], Trails B [21], or Block Design of the WAIS-III [19]. Finally, CN elderly subjects evidenced a CDR score of 0 as scored by the clinician and no memory or nonmemory measures that scored 1.0 SD or below expected levels. A full description of the characteristics of these samples is described below.

## 3. Procedures

The Miami Prospective Memory Test (MPMT [22]) was designed to evaluate time-related and event-related prospective memory ability of older adults in a clinical setting. The MPMT Event-Related Task involves setting a timer with a loud ring for 30 minutes. After the bell rings, the subject is asked to pick up an envelope that is located on the desk in view and close to the examiner. The subject is then asked to open the envelope and select from a number of different denominations (currency and change), a $5 dollar bill, which is supposed to be handed to the examiner, and a $10 dollar bill, which the participant should give to themselves. Subjects are scored on intention to respond by their reaction to the loud bell, accuracy (selecting the correct monetary denomination for the examiner and the participant), and need for cues (the degree to which the participant requires prompts from the examiner). Each of these three elements are scored 0–3 with a maximum possible score of 9 points.

An example of the scoring system is as follows.

*(I) Intention to Perform*

 
Score = 3: spontaneously takes envelope when the oven timer bell goes off.
 
Score = 2: does not take envelope but gives indication verbally that he/she needs to do something in response to signal (e.g., “I know I am supposed to do something but I cannot remember what it is”).
 
Score = 1: provides a nonspecific, nonverbal response to signal (e.g., looks around the room, looks at area where the bell rang, startle response).
 
Score = 0: provides no response to signal.

*(II) Accuracy of Response*

 
Score = 3: subject correctly gives the examiner the $5 dollar bill and gives to self the $10 dollar bill.
 
Score = 2: subject correctly selects the $5 and $10 dollar bills but do not use them correctly (e.g., gives the examiner the $10 dollar bill and gives to self the $5 dollar bill).
 
Score = 1: subject selects the $5 or $ 10 dollar bill and gives it to self or examiner. Assign a score of 1 regardless of which one (i.e., the $5 or $10) is given to whom (i.e., self or examiner).
 
Score = 0: none of the above. Some alternatives are as follows:
  - subject does not select the $5 or $10 dollar bill but rather selects other denominations or only selects coins;
  - subject selects $5 or $10 dollar bill but does not take any of these for self nor does he/she give it to the examiner;
  - subject does not select any money from the envelope (e.g., gives envelope to examiner with all the money in it).

*(III) Need of Reminders*. When the timer goes off, allow a 60-second grace period for the subject to initiate a response.

If subject has not initiated a response within 60 seconds, initiate provision of hierarchical cues as follows by saving.

(Cue  1) “You were supposed to do something when the timer went off. Do you know what it was?”

Please select one of the following based on subject's response to cue:

 was able to complete the task without further cues or errors;
 has some idea (i.e., based on verbal comments or actions) that the response had to do with the envelope and the money;
 has some idea (i.e., based on verbal comments or actions) that the response had to do with the envelope or the money.
(Cue  2) Subject responds incorrectly or his/her response does not include grabbing the envelope on the desk, say **“**you were supposed to do something with this envelope (show envelope to subject). Do you know what it was?”
(Cue  3) If response does not include a description of giving money to examiner or self, say “you were supposed to do something with the money in this envelope (show envelope to subject). Do you know what it was?”
 Score = 3: no reminder is needed.
 Score = 2: needs  *only* one of the above reminders.
 Specify reminder given:_______________________
 Score = 1: needs two of the above reminders. Specify reminders given:*_______________________*
 Score = 0: needs all three reminders. Assign a score of 0, regardless of whether the response to the third reminder was accurate or not.

The MPMT Time-Related Task (Trial 1) involves setting a large analogue clock behind the examiner who is administering nonmemory cognitive tests. The clock is initially set for 8:00 and the subject is requested to interrupt the examiner at 8:15 and request an envelope with five cards with different numbers. The participant is required to give the examiner a card with a specific number and herself a card with another specific number. Subjects are scored on intention to respond by asking for the card when the clock reaches 8:15, accuracy (selecting the correct card for both the examiner and the participant), and need for cues (the degree to which the participant requires prompts from the examiner). Each of these three elements are scored 0–3 with a maximum possible score of 9 points.

The MPMT Time-Related Task (Trial 2) employs the same paradigm as the MPMT Time-Related Task (Trial 1) except that the time interval is increased to 30 minutes. Subjects are scored on intention to respond by asking for the card when thirty minutes elapses, accuracy (selecting the correct card for both the examiner and the participant), and need for cues (the degree to which the participant requires prompts from the examiner). Each of these three elements are scored 0–3 with a maximum possible score of 9 points.

The total possible number of total points for the MPMT is 27 points.

## 4. Results

### 4.1. Test-Retest Reliability

Fourteen elders (8 males and 6 females) age 67 to 98 years (mean age = 78.1; SD = 7.6 years) had a Global Clinical Dementia Rating Scale (CR) 0f .5 and were diagnosed with amnestic MCI (aMCI) by Petersen's criteria [16]. Twelve individuals spoke English as their primary language and 2 spoke Spanish as their primary language. The mean MMSE scores for this group were 27.9 (SD = 1.6) and the suspected clinical etiological diagnosis based on clinical evaluation for 86% of these individuals was MCI attributable to Alzheimer's Disease (AD [23]). In one case, an individual was diagnosed with MCI attributable to a vascular etiology, and in another case, the individual had suspected Diffuse Lewy Body disease. All aMCI subjects were administered the MPMT on two occasions within a 9-week interval (mean 5.6 weeks; SD = 1.8 weeks). Test-retest comparisons were conducted for the Total MPMT Event Score, Total MPMT Time Score, and Total MPMT Score. High, statistically significant test-retest reliabilities based on two-tailed Pearson Product Moment Tests were obtained for Total MPMT Event Score (*r* = .58; *P* < .03), PMT Time Score (*r* = .55; *P* < .05) and Total MPMT Score (*r* = .65; *P* = .02).

### 4.2. Discriminative Validity

We performed discriminative validity studies on 71 aMCI participants (41.2% females: 60.3% English speakers) who met Petersen's [16] criteria for aMCI and had a CDR global score of .5. All subjects had memory scores at 1.5 SD below expected levels on one or more of the Fuld-OME, Delayed Memory for Passages of the WMS-III, or Delayed Visual Reproduction of the WMS-III. The age range of these patients was between 67 and 98 years of age (mean = 77.9, SD = 6.4) with the average MMSE scores ranging from 23 to 30 (mean = 26.2 SD = 2.0).

We also assembled 17 participants (52.9% female: 53.9% English speakers) who met Petersen's [16] criteria for nonamnestic MCI and had a CDR global score of .5. All subjects had nonimpaired scores on memory measures described above but scored 1.5 SD or lower on one or more nonamnestic measures such as letter fluency, category fluency, Trails B, or Block Design of the WAIS-III. The age range of these patients was between 59 and 86 years of age (mean = 76.6; SD = 7.0) with the average MMSE scores ranging from 23 to 30 (mean = 26.5 SD = 1.8).

Finally, we recruited 133 normal elderly (NE) participants (69.6% females: 67.7% English speakers) who had a CDR score of 0 as scored by the clinician and no memory or nonmemory measures that scored no less than 1.0 SD below expected levels. The MMSE scores for this group were 26 or greater and the age range of these patients was between 65 and 93 years of age (mean = 76.0; SD = 5.2) years. Average MMSE scores ranged from 26 to 30 (mean = 28.3; SD = 1.2).

There were no statistically significant group differences found for age (*F *(2,218) = 2.76; *P* < .07) although there were statistically significant differences in educational attainment (*F *(2,217) = 12.79; *P* < .001). Post-hoc Tukey's HSD tests revealed that naMCI subjects had lower levels of educational attainment than the other diagnostic groups. There also were significant group differences for MMSE scores (*F *(2,2517) = 41.44; *P* < .001). Tukey HSD post-hoc tests revealed that cognitively normal participants had higher average MMSE scores than both aMCI and naMCI groups. Chi-square analyses revealed significant differences between groups with regard to gender (*χ* ^2^ (df = 3) = 14.10; *P* < .04). There was a higher proportion of males in the aMCI group relative to normal elderly group. There were no differences in proportion of English speakers versus Spanish speakers in different diagnostic groups.

We examined discriminative validity in this study by the performance of the aMCI cohort and other diagnostic groups with normal elderly control subjects. As indicated in Table 1, the results of ANOVA models with post-hoc Tukey's HSD tests indicated that all aMCI participants scored lower on all indices of the MPMT as compared to normal elderly subjects and naMCI subjects. NE subjects and naMCI participants had equivalent mean scores on all MPMT measures.

**Figure 1:**
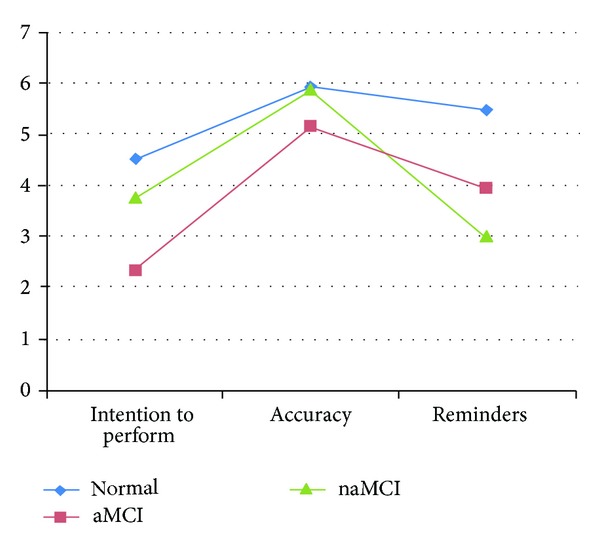


Diagnostic groups were then compared on their scores on intention to perform, accuracy, and need for prompts/reminders by combining performance in these specific domains across the Event-Related prospective memory task and the first Time-Related Prospective Memory Test. This was accomplished using a 3 × 3 (Measurement Type by Diagnostic Group) mixed model repeated measures design. Measurement Type (intention to perform, accuracy, or need for reminders) served as the within subject repeated measures, while the Diagnostic Group (normal elderly, aMCI, or naMCI) served as the between group factor. Results indicated a statistically significant effect for Group (*F* (2,216) = 48.01; *P* < .01), Measure Type (*F* (2,432) = 12.77; *P* < .01) and the Group by Measure Type Interaction (*F* (2,432) = 48.01; *P* < .01). In general, the aMCI group scored more poorly on all types of MPMT measures and overall for all groups. Intention to perform was the most impaired task followed by need for reminders and then accuracy as assessed by the Sidak post-hoc examination of means. The statistically significant interaction term in Figure 1 depicts the discrepancy between the poor intention to perform score and the higher accuracy score, which was particularly pronounced for aMCI patients as compared to the other study groups.

As a further step, we determined the extent to which different MPMT measures could distinguish between aMCI and NE subjects using logistic regression. There were statistically significant results when the diagnostic groups were compared on MPMT Total Event Scores (Wald = 44.2; *P* < .001; Sensitivity = 74.6%; Specificity = 72.7%; overall =  73.4%); PMT Time 1 Scores (Wald = 22.8; *P* < .001; Sensitivity = 37.1%; Specificity = 92.4%; overall = 73.3%); PMT Time 2 Scores (Wald = 17.90; *P* < .001; Sensitivity = 31.9%; Specificity = 97.0%; overall = 74.6%); Total PMT Score (Wald = 39. 29; *P* < .001; Sensitivity = 49.3%; Specificity = 90.9%; Overall = 76.6%) and Event PMT + Time 1 PMT Score (Wald = 41.67; *P* < .001; Sensitivity = 48.2%; Specificity = 90.2%; overall = 75.7%).

As depicted in Table 2, when using optimal cut-off scores derived from the aforementioned logistic regression models (which maximized total correct classification), there were statistically significant group differences in the percentage of NE, aMCI, and naMCI subjects who were classified as impaired across all Event-Related, Time Related, and Combined Event-Related and Time Related conditions (*P* < .001). For example, using a cutoff of 12 (out of 18 points) for the combined Event-Related and Time 1 measures, impairments were observed in 48.6% of aMCI and 29.4% of naMCI and 10.0% of normal elderly participants (*χ* ^2^ = 37.45; *P* < .001).

Post-hoc chi-square analyses revealed that group differences in proportions was largely due to statistically significant differences in impairment between NE and aMCI groups. However, post-hoc 2 × 3 Fisher's exact test chi-square tests revealed that relative to NE subjects, naMCI participants evidenced greater impairment on the MPMT Event-Related measure (*P* < .05) and MPMT Event + MPMT Time 1 measure (*P* ≤ .04). The MPMT Time 1 measure approached statistical significance (*P* < .06).

## 5. Discussion

This study represented a first attempt to distinguish aMCI, naMCI, and normal elderly subjects using a novel prospective memory test (MPMT). The MPMT paradigm that was employed is unique because (a) the measure assesses both time and event-related prospective memory and (b) separate scores are provided for the intention to perform, accuracy of responses, and need for reminders. This allowed for an examination of specific components of prospective memory that may be compromised in at-risk older adult populations.

The MPMT showed high test-retest reliability among carefully diagnosed patients with aMCI. More importantly, aMCI subjects uniformly exhibited greater deficits on each and every time and event-related subtest of the MPMT relative to naMCI subjects and cognitively normal elders. Further, almost a third of naMCI patients exhibited prospective memory deficits despite the lack of impairment on standard measures of memory. Almost 50% of patients with aMCI evidenced prospective memory impairment suggesting heterogeneity among MCI patients as a whole. This was demonstrated by some patients exhibiting isolated prospective memory deficits, other patients showing isolated retrospective memory deficits, while other patients demonstrated both types of impairments.

Interestingly, for MCI patients, the MPMT domains that were most impaired were intention to perform, followed by need for reminders with subjects scoring higher on accuracy of responses. This is consistent with an emerging body of literature suggesting that the ability to remember an intended action may be as sensitive or more sensitive than retrospective memory alone in MCI patients (see [24]).

Prospective memory largely depends upon the integrity of multiple cognitive abilities associated with frontal and temporolimbic systems [25], including working memory, executive functions, retrospective memory, and information processing speed (e.g., [26]). Thus, impairments on MPMT tasks are likely related to a breakdown in functional subsystems that are not limited to the hippocampal and entorhinal cortex difficulties observed in early AD.

Despite its importance and relevance to a large number of clinical complaints in the elderly, time and event related prospective memory measures are not routinely administered in clinical evaluations, although there is an increasing consensus that PM is a useful construct that should be employed in standard neuropsychological evaluations of MCI [27].

Our study is unique in that we studied both aMCI and naMCI patients. However, it is acknowledged that test-retest reliabilities were conducted on a relatively modest number of aMCI patients and the sample of naMCI participants available for discriminative validity studies was considerably less than the aMCI and NC groups. Clearly, future studies would benefit from larger numbers of subjects to establish generalizability to these and other patient groups. Further investigation into prospective memory impairments and dissociating retrospective memory from the executive components of specific tasks should have heuristic and clinical relevance that can better our understanding of specific memory systems compromised in early neurodegenerative disease.

## Figures and Tables

**Table 1:** Mean comparisons of different diagnostic groups on different PMT measures.

	Normal elderly (*N* = 132)	NaMCI (*N* = 17)	aMCI (*N* = 71)	ANOVA *F*
PMT Event 1	7.56^b^ (SD = 2.0)	6.71^b^ (SD = 2.3)	4.63^a^ (SD = 2.5)	41.44***
PMT Time 1	8.36^b^ (SD = 1.2)	7.82^b^ (SD = 1.6)	6.73^a^ (SD = 2.7)	18.43***
PMT Time 2	8.48^b^ (SD = 1.1)	8.53^b^ (SD =0 .7)	7.12^a^ (SD = 2.5)	16.33***
PMT Total	24. 45^b^ (SD = 2.8)	23.06^b^ (SD = 3.0)	18.52^a^ (SD = 5.8)	48.87***
PMT Event 1 + PMT Time 1	15.92^ b^ (SD = 2.5)	14.53^ b^ (SD=3.1)	11.43^a^ (SD = 4.0)	48.01***

*Note*. (1) Following a statistically significant test *F* value post-hoc tests were conducted using Tukey's HSD procedure. (2) Means with different alphabetic superscripts are statistically significant at *P* < .05 by Tukey's HSD procedure. (3) aMCI: amnestic MCI; NaMCI: nonamnestic MCI; (4) ***P < .001.

**Table 2:** Comparative percentages of impairment on different MPMT tasks.

	PMT Event 1 Cutoff ≤ 5	PMT Time 1 Cutoff ≤ 6	PMT Time 2 Cutoff ≤ 6	PMT Total Cutoff ≤ 12	PMT Event 1 + PMT Time 1 Cutoff ≤ 20
Normal elderly (*N* = 133)	26.9% impaired	7.7% impaired	3.1% impaired	10.0% impaired	9.2% impaired
aMCI (*N* = 71)	74.6% impaired	37.1% impaired	31.9% impaired	48.6% impaired	49.3% impaired
NaMCI (*N* = 17)	52.9% impaired	23.5% impaired	0.0% impaired	29.4% impaired	23.5% impaired
χ^2^ (df = 2)	42.88***	26.57***	39.85***	37.45***	40.63***

*Note*. (1) ***P < .001 by the chi-square procedure on percentages compared for each MPMT measure. (2) aMCI: amnestic MCI; NaMCI: nonamnestic MCI.
